# Transactions of the Homœopathic Medical Society of Pennsylvania

**Published:** 1888-02

**Authors:** 


### TRANSACTIONS OF THE HOMŒOPATHIC MEDICAL SOCIETY OF PENNSYLVANIA. Twenty-third Annual Session.

This volume contains some very useful articles. Especially to be commended are the repertories of urinary and heart symptoms arranged by Drs. Theodore J. Gramm and E. R. Snyder. These are made up of symptoms taken from Hering's *Condensed Materia Medica.* They together fill about one hundred and twenty-four pages, and are *very* good.

More work of this kind is promised in the future by this bureau. All such work is doing good service for Homœopathy.

